# Triggering Degradation of Cellulose Acetate by Embedded Enzymes: Accelerated Enzymatic Degradation and Biodegradation under Simulated Composting Conditions

**DOI:** 10.1021/acs.biomac.3c00337

**Published:** 2023-06-22

**Authors:** Naba Kumar Kalita, Minna Hakkarainen

**Affiliations:** Department of Fibre and Polymer Technology, KTH Royal Institute of Technology, Teknikringen 58, 100 44 Stockholm, Sweden

## Abstract

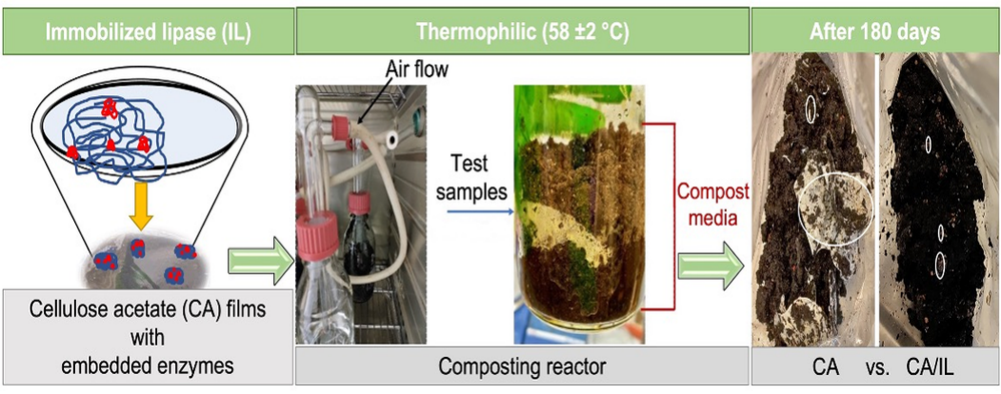

A green strategy that significantly accelerates the biodegradation rate of cellulose acetate (CA) by triggering deacetylation was demonstrated. Lipase isolated from *Candida rugosa* was immobilized on CA particles (immobilized lipase (IL)) by a physical entrapment method and further incorporated in CA films. After 40 days of aging in contact with external enzymes (lipase and cellulase), the number-average molecular weight (*M*_n_) of CA/IL 5% decreased by 88%, while the *M*_n_ of CA only exhibited a 48% reduction. Fourier transform infrared and nuclear magnetic resonance spectroscopy of CA/IL 5% indicated significant deacetylation, which was further supported by the decrease of the water contact angle from 59 to 16°. These drastic changes were not observed for CA. Similar differences in the degradation rate were observed during aging under simulated composting conditions. After 180 days of simulated composting, traces of CA/IL 5% were barely observable, while large pieces of CA still remained. This could open the door to modified lignocellulose materials with retained biodegradability, also reducing the requirements for the degradation environment as the process is initiated from inside of the material.

## Introduction

Biodegradation of potentially biodegradable plastics typically requires a specific environment, which means it is difficult to guarantee complete biodegradation if the end-of-life environment is unknown.^1−3^ Synthetic biodegradable plastics contain, in most cases, ester moieties and include, e.g., poly(lactic acid) (PLA), poly(ε-caprolactone) (PCL), and poly(butylene terephthalate-*co*-adipate) (PBAT). Esterification, especially acetylation, is also used to modify biopolymers to decrease their hydrophilicity and to introduce thermoplastic properties. Cellulose acetate (CA) and other cellulose esters are, thereby, among the most common commercial cellulose derivatives. Native cellulose can be degraded in most natural environments, although the rate greatly depends on the conditions in the specific environment.^2^ The biodegradation and enzymatic degradation of cellulose esters have been extensively investigated in different environments.^4−8^ Unfortunately, the susceptibility of cellulose esters to degradation typically significantly decreases as the degree of substitution and the size of the ester substituents increase.^9,10^ For CA, the deacetylation reaction, releasing acetic acid and finally leading to the regeneration of cellulose, is typically the rate-determining step.^2^

Various approaches have been evaluated to trigger the degradation and deacetylation of CA. As an example, to increase the degradation in natural environments, photocatalysts have been embedded in materials, aiming to initiate the environmental degradation process when the materials are subjected to UV light from the sun.^11^ Other approaches require pretreatments of the materials such as immersion in various salt solutions to facilitate the subsequent biodegradation process in, e.g., compost.^5,12,13^ These approaches are promising, but the degradability still remains an issue, as a suitable pretreatment before composting or specific conditions, such as contact with sunlight, are needed to initiate the degradation process. Recently, it was shown that self-degradation of polyesters, such as PLA^14^ and PCL,^15^ can be initiated by embedding immobilized enzymes in the polymer matrix. Immobilization also increased the thermal stability of the enzymes so they could be processed using extrusion or thermoforming.^15^ As an example of the effectivity of this enzyme-embedding approach, 78% weight loss was observed for poly(l-lactic acid) (PLLA) films with embedded enzymes 96 h after incubation in Tris–HCl buffer, while no weight loss was observed for the plain PLLA films during the same time period. The degradation of PLLA films incubated in solution with external enzymes also proceeded significantly slower compared to the films with embedded enzymes.^14^ Three-dimensional (3D) printing of enzyme-embedded PCL was also demonstrated.^16^ This work further supported the faster degradation of the materials with embedded enzymes compared to the degradation with externally applied enzymes. Furthermore, the degradation rate could be tuned by concentration of the embedded enzyme with 5.2 and 100% weight losses for the films with 0.1 and 5.0% Amanose lipase, respectively. A potential negative impact of the embedded enzymes can be a reduction of material properties. This was resolved by substituting microsized embedded enzymes by nanodispersed enzymes,^15^ which also increased the enzymatic degradation rate likely due to the increased contact area.

The degradability or not of cellulose derivatives, such as CA, remains a point of discussion.^17^ As already stated above, it is well-known that deacetylation is the rate-determining step, leading finally to regenerated cellulose, which is degradable in common natural environments.^18^ Embedding enzymes that can catalyze ester hydrolysis in aliphatic polyesters was recently shown to be an effective route to trigger rapid degradation.^14,15^ We hypothesized that a similar approach could be applicable to trigger the degradation of cellulose esters, such as CA, i.e., embedding lipase capable of deacetylating CA, could be the key to ensuring degradation of CA in common natural environments and in compost. Furthermore, we aimed to immobilize the enzyme on CA particles that would be further incorporated in the CA polymer matrix. In this way, no extra components are added, and we avoid commonly used nondegradable entrapment matrices such as polyacrylates or silica, further avoiding the release of potentially persistent microparticles. The effectiveness of this approach was evaluated by investigating the enzymatic degradation and degradation under simulated composting conditions of CA films with embedded enzymes.

## Experimental Section

### Materials

Cellulose acetate (CA; 30 kDa, DS = 2.2 from NMR analysis^5^), lipase from *Candida rugosa*, cellulase from *Aspergillus niger*, and 4-nitrophenol butyrate (*p*NPB) were purchased from Merck. Acetone, ethyl acetate, phosphate-buffered solution (PBS, pH 7.4), and dimethyl sulfoxide (DMSO) were of technical grade and purchased from VWR. Compost was prepared according to ASTM standard D 5338-21, and biomass was purchased from the local market.

### Immobilization of Lipase by Physical Entrapment in CA

CA (50 mg mL^–1^) was stirred for 6 h in acetone. Simultaneously, lipase (3–5 mg mL^–1^) was stirred for 2 h in 0.1 mM PBS buffer (pH 7.4). The CA solution was then mixed with the buffer solution with enzymes. The mixture was poured into dehydrated acetone and stirred for an additional 4 h at 450 rpm, after which it was washed with water up to four times or until the washing out of the unbound enzymes was confirmed by an ultraviolet–visible (UV–vis) spectrophotometer at 280 nm. The enzyme solutions before and after immobilization were collected, and the enzyme concentration was determined by a UV–vis spectrophotometer with the help of a calibration curve at 280 nm. The collected immobilized enzyme was freeze-dried for 72 h and stored at 4–6 °C.^19^

### Immobilization Ratio

The immobilization ratio I was calculated by eq 1
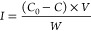
1where *C*_0_ is the initial lipase concentration, *C* is the free lipase remaining after immobilization (mg mL^–1^), *V* is the volume of the lipase solution added into the column at the initial time (mL), and *W* is the weight of the dried immobilization matrix with enzyme loading (mg). The immobilization ratio gives the ratio of enzymes in the immobilized matrix.

### Enzyme Activity (Immobilization Efficiency)

Enzyme activity was calculated according to eq 2.
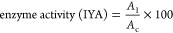
2

### Michaelis–Menten (MM) Kinetics

The enzymatic activities of free lipase and immobilized lipase (IL) were evaluated by Michaelis–Menten kinetics. 4-Nitrophenol butyrate, of concentration 5–10 μmol mL^–1^, was used as a substrate. The enzyme concentration was kept constant (2 μg mL^–1^). Eq 3 was used to evaluate the enzyme kinetic parameters measured at 415 nm.
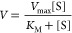
3where *V*_max_ is the maximum reaction rate attained at infinite substrate concentration, *S* is the substrate concentration (mg mL^–1^), and *K*_M_ is the Michaelis–Menten constant (mg mL^–1^). Lineweaver–Burk (LB) and Michaelis–Menten (MM) plots were used to establish the kinetic parameters.^20^

### Thermal Stability of the Immobilized Enzymes

Enzymatic activity was evaluated by following the hydrolysis of 4-nitrophenol butyrate (*p*NPB) with a concentration of 5 μmol mL^–1^ by UV–vis spectroscopy at different temperatures in a PBS of pH 7.4. The temperatures were set at 25, 40, 60, 80, and 100 °C for IL and at 25, 40, and 60 °C for free lipase 25–40 °C since lipase is known to show the best activity at ∼37 °C.^21^ The enzyme concentration was kept constant (2 μg mL^–1^) for both IL and free lipase.

### Preparation of CA Films with and without Embedded Enzymes

CA (20 mg mL^–1^) with or without embedded enzymes was prepared by stirring at 40 °C for 24 h and solution casting using a binary solvent of 70/30 acetone/ethyl acetate. The prepared films were named CA (100 wt % CA), CA/IL 3% (3 wt % immobilized lipase/97 wt % CA), and CA/IL 5% (5 wt % immobilized lipase/95 wt % CA).

### Characterization

#### Confocal Laser Scanning Microscope (CLSM)

To probe enzyme distribution in the films, lipase was fluorescent-labeled (FL) by NHS–fluorescein (5/6-carboxyfluorescein succinimidyl ester) following the manufacturer’s procedure; a 460–490 nm excitation wavelength was used to take the fluorescence microscopy images using an LSM 510, Zeiss. The samples were mixed with a fluorescamine solution (50 mg mL^–1^ in acetone) for 3 min to form a highly fluorescent product through the reaction between the primary amines in proteins and the fluorescamine.^19^

#### Fourier Transform Infrared (FTIR) Spectroscopy

Fourier transform infrared spectra of the free lipase, CA, CA/IL 3%, and CA/IL5% films before and after degradation under different conditions were obtained by a PerkinElmer Spectrum 2000 FTIR spectrometer using the attenuated total reflectance (ATR) mode. A total of 16 scans were recorded in the wavenumber range of 600–4000 cm^–1^.^5^

#### Nuclear Magnetic Resonance (NMR) Spectroscopy

^1^H NMR spectra (64 scans) were collected on a Bruker Avance 400 MHz spectrometer. In a 5 mm diameter NMR tube, the samples (∼5 mg) were dissolved in 0.7 mL of deuterated dimethyl sulfoxide (DMSO-*d*_6_). The DS for acetylation of CA was estimated from the obtained spectra using eq 4.^5^ The ^1^H NMR spectra of CA and enzyme-embedded CA-based films exposed to various degradation conditions exhibited spectral lines in the ring proton region at 5.2–3.6 ppm and for the acetyl groups at 1.80–2.15 ppm.
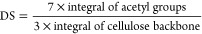
4

#### Ultraviolet–Visible (UV–Vis) Spectroscopy

The absorption values of immobilized lipases, free lipase, and Michaelis–Menten kinetics were measured by a Shimadzu UV-2550 UV–vis spectrophotometer. The measurements were carried out using a quartz cuvette with a width of 1 cm at different wavelengths.

#### Size-Exclusion Chromatography (SEC)

The number- and weight-average molecular weight (*M*_n_, *M*_w_) and dispersity (*Đ*) of CA, CA/IL3%, and CA/IL5% before and after degradation under different conditions were determined by SEC. The analyses were performed in DMSO/0.5 wt % LiCl at 23 °C using an Agilent size-exclusion chromatograph equipped with a Knauer 2320 refractometer index detector and two PL Gel columns (MIXED-D and 103A). Before analysis, the samples were dissolved in DMSO (3 mg mL^–1^) and 20 μL of the solutions was injected into the SEC columns using a flow rate of 1 mL min^–1^. Monodisperse pullulan standards were used for the calibration.

#### Thermal Gravimetric Analysis (TGA)

A Mettler Toledo TGA/SDTA 851e was utilized for the thermogravimetric analysis of the free lipase and CA, CA/IL3%, and CA/IL5% films before and after degradation under different conditions. 2–10 mg of each sample was placed into a 70 μL alumina cup. The samples were then heated at the rate of 10 °C min^–1^ from 30 to 700 °C with a N_2_ flow rate of 50 mL min^–1^.

#### Water Contact Angle (WCA) Measurements

Water contact angles of the films before and after degradation were measured using a Theta Lite, Biolin Scientific goniometer. 4 μL water droplets were utilized to measure the contact angle at 25 °C.

#### Scanning Electron Microscopy (SEM)

SEM images were acquired by an ultrahigh-resolution field emission scanning electron microscopy (FESEM) Hitachi S-4800. The samples were sputter-coated (Cressington 208HR Sputter Coater) with platinum/palladium (Pt/Pd) at 2–4 nm thickness prior to the analysis before and after degradation under different conditions.^22^

#### Enzymatic Degradation and Degradation under Composting Conditions

The susceptibility of CA, CA/IL 3%, and CA/IL5% to enzymatic degradation in an aquatic solution and biodegradation under simulated composting conditions was evaluated. The enzymatic degradation was evaluated in PBS (pH 7.4) containing 10 mg/100 mL lipase and 10 mg/100 mL cellulase and replenishing them on every 7th day. Samples (1 cm × 1 cm) were prepared in triplicate. The sample weight was measured before placing them in the PBS (20 mL) at 37 °C inside an incubator.^15^ The materials were also subjected to biodegradation under a simulated composting condition at 58 ± 2 °C. For the composting study, 500 mL glass vessels were used where 200 g of compost and 10 g of the prepared test samples were used. The vessels were supplied with air continuously throughout the degradation period in order to maintain the aerobic composting condition and were replenished with distilled water every 7th day for maintaining the required relative humidity. The mature compost consisted of animal manure and food waste. The test samples were characterized by SEM, SEC, FTIR, NMR, and WCA before and after degradation.^3,7^

#### Hydrolytic Stability of the Prepared Test Samples

The hydrolytic stability of CA and CA/IL 5% films was evaluated by subjecting the films to pure water for 180 days at 37 °C. The solution was replenished with fresh water every 7th day, and the test samples were characterized by SEM, SEC, FTIR, NMR, and WCA before and after hydrolytic aging.

## Results and Discussion

Enzymes were physically entrapped in CA particles, and the CA immobilized enzymes were further embedded in CA films (DS = 2.2) to evaluate the ability of the entrapped enzymes to catalyze the degradation of CA under different conditions.

### Design of the Polymer–Enzyme Matrix for Enzyme Entrapment

**Figure 1:**
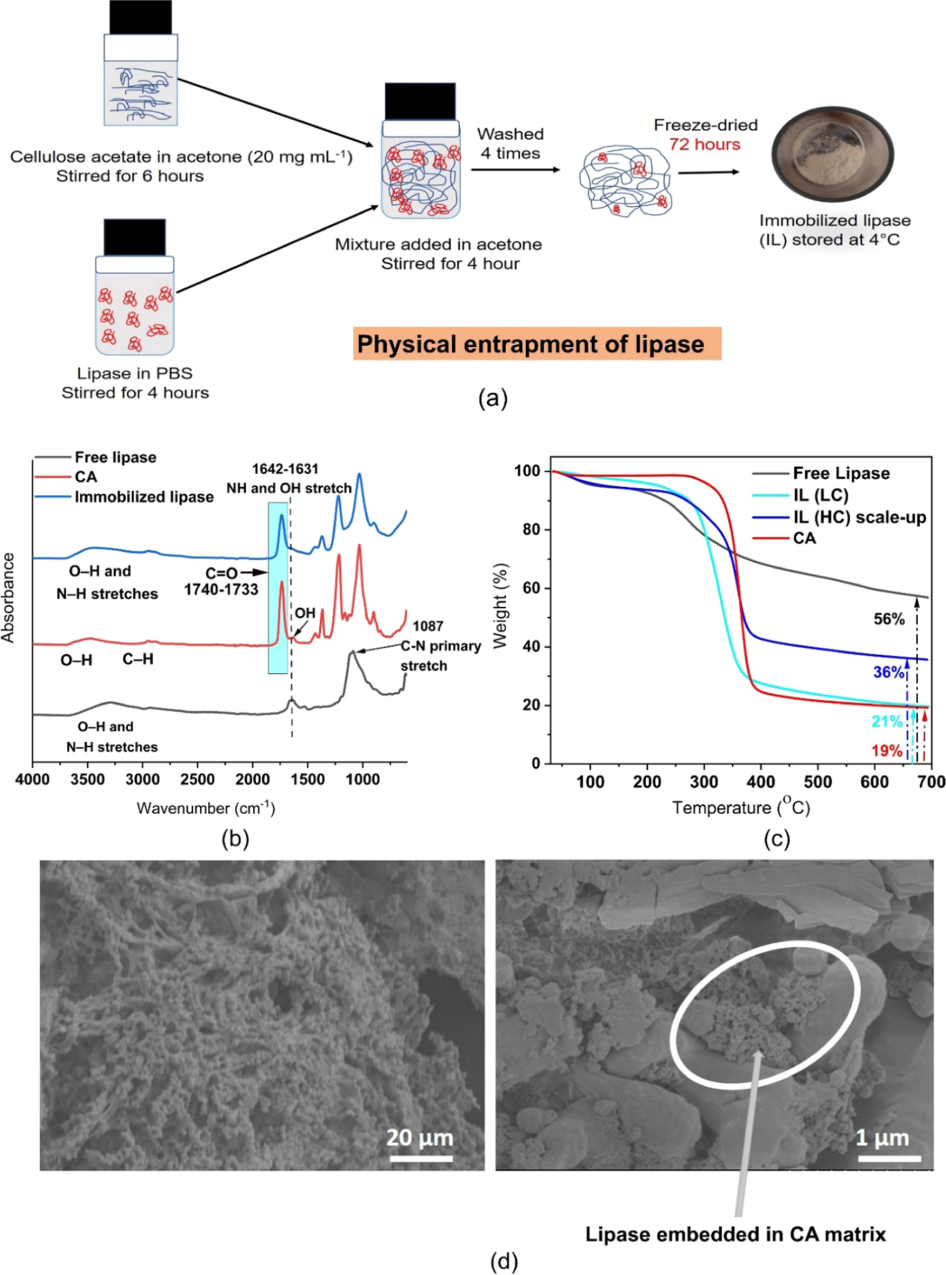
(a) Schematic illustration showcasing the physical entrapment of lipase into CA. (b) FTIR spectra of IL, lipase, and CA. (c) TGA thermograms of free lipase, IL, and CA. (d) SEM images demonstrating lipase immobilized in CA through physical entrapment.

Ideally, the immobilization matrix for the enzymes would be the same as the polymer matrix used to prepare the films to minimize the number of components and to keep the biobased nature of the materials. Therefore, we evaluated CA as the immobilization matrix for the physical entrapment of the lipase. Figure 1a depicts a schematic illustration of the physical entrapment method. The lipase together with CA as the entrapment matrix is hereafter denoted as immobilized lipase (IL). FTIR confirmed the increased peak broadness for IL in comparison to neat CA (Figure 1b). The OH band observed at 3600–3300 cm^–1^ for CA was broadened to a lower wavenumber due to the introduction of lipase NH groups. The intensity of acetyl group vibrations at around 1240 and 1740 cm^–1^ in the immobilized lipase matrix decreased compared to the cellulose C–O–C stretch, which might indicate that some deacetylation of CA took place already during the immobilization process. The broadening of the peak observed at 1650–1550 cm^–1^ in the immobilized lipase matrix, compared to the OH peak in neat CA, is due to the introduction of secondary amine groups.

The higher thermal stability of the immobilized enzyme was further established by comparing the activity of the immobilized enzyme (IL) at 25, 40, 60, and 100 °C and the free lipase at 25, 40, and 60 °C in hydrolyzing pNPB. Figure S1 supports the fact that the enzymes were effectively immobilized. Free lipase showed some activity at 25 °C, while IL scarcely revealed any activity at that temperature. IL began exhibiting activity at 40 °C, and this was intensified at 60 °C, and then decreased when the temperature was raised additionally to 100 °C. The activity of free lipase, however, peaked already at 40 °C, while it reduced already at 60 °C, falling to approximately 0.08 from 0.99. The IL thus demonstrated some activity up to 100 °C, while the free lipase was only active between 25 and 40 °C. Earlier studies reported that the same lipase had the best activity at 37 °C^21^ in PBS.

Thermogravimetric analysis (Figure 1c) revealed that the IL was more stable toward thermal degradation compared to the neat free lipase.^23^ This is likely connected to the stabilizing effect of the more thermally stable CA as an entrapment matrix. In the TGA thermograms, we could also observe that free lipase loses weight significantly after reaching a temperature of 200 °C, which could be related to the release of strongly bound water that is attached to the enzyme.^24^ This water is necessary for the catalytic activity of the enzyme.^25,26^ This was not observed as clearly for IL, which could indicate an enhanced capacity to bind water after immobilization and entrapment in the CA matrix. This would be beneficial for the structural flexibility and, consequently, the activity of the immobilized enzyme.^27^ In the case of IL (lower concentration (LC) of enzyme, 30 mg mL^–1^), the TGA curve resembled a lot the TGA curve of CA, especially the amount of residue was similar (21 versus 19% for CA), although the degradation was initiated earlier. For IL (higher concentration (HC), 50 mg mL^–1^), the amount of residue increased to 36%, which is intermediate between CA and free lipase (56%). This also supports the role of the higher initial lipase concentration (HC) to increase the amount of lipase entrapped within the CA matrix. The immobilization also limits the thermal motions of the enzymes, preventing denaturation and boosting their thermal stability. The large residue observed is inherent to free lipase and similar to that of earlier reported results.^26,28^ The physical entrapment of lipase in CA (Figure 1d) can increase the thermal stability and help retain the catalytic activity, which can be further tuned by increasing the concentration of lipase during immobilization. The immobilization ratio and enzymatic activity of the prepared IL are presented in Table S1 and Figures S2 and S3, respectively. For all of the CA-based films, the used IL was HC. This higher concentration was selected for the expected higher effectivity to catalyze the CA degradation.

### Enzyme Kinetics to Understand the Effect of Immobilization

Michaelis–Menten kinetics was utilized to explain the velocity and to draw a mechanism of enzyme-catalyzed reactions before and after immobilization. The generated adsorption data sets were analyzed using PRISM software.

**Figure 2:**
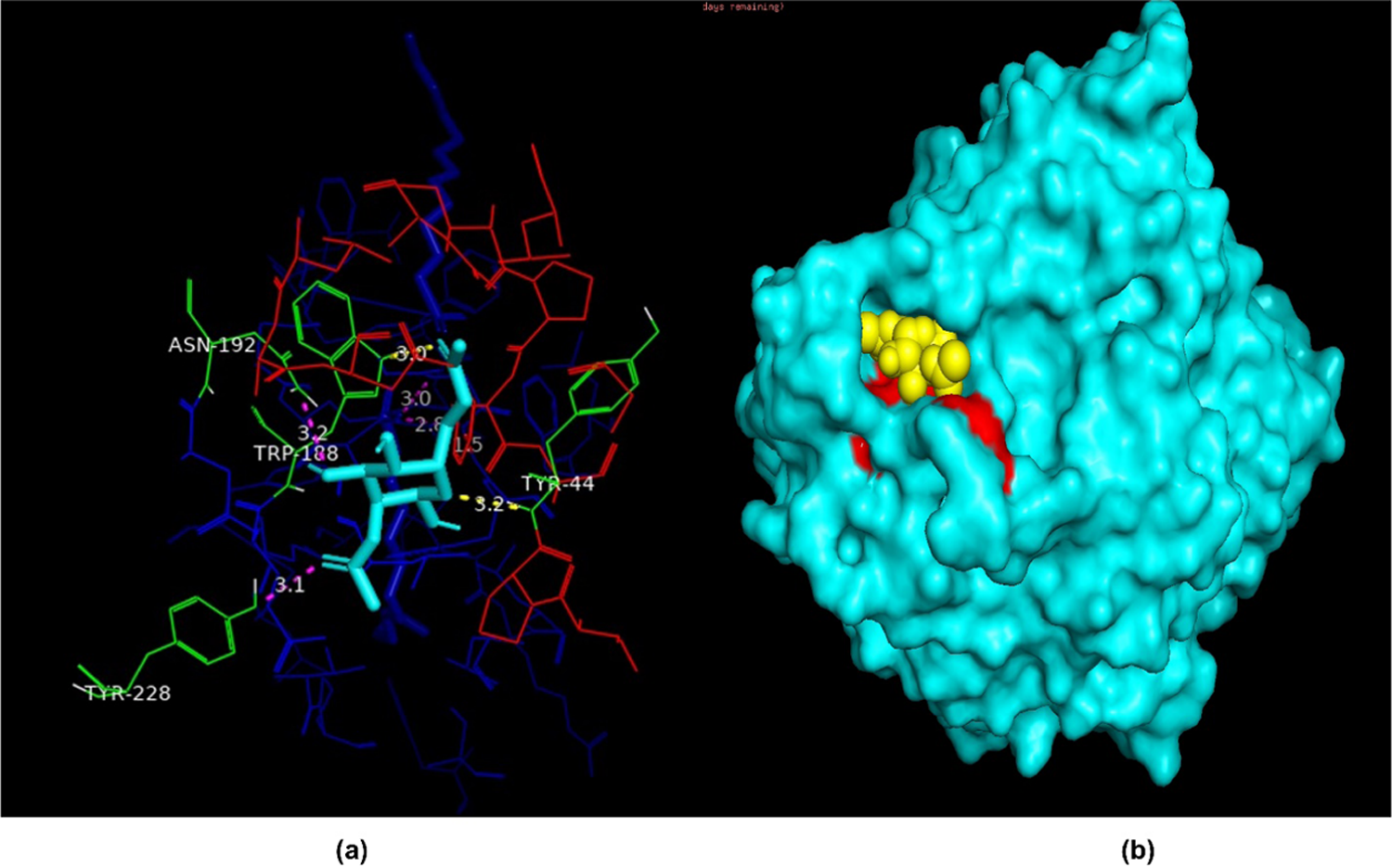
(a) Stick representation (display of 20 Å sphere) of IL showing two catalytic sites. Cyan represents the CA ligand, blue represents nonpolar interactions, and red represents polar interactions having asparagine (Asn), tyrosine (Tyr), and serine (Ser) residues showing their catalytic activity. A docking score of −6.5 and an root-mean-square deviation (RMSD) score of 0.61 confirm the good affinity. A polar bond length of 3–3.8 Å was observed. (b) Surface representation of IL. Cyan represents nonpolar interactions, red represents polar interactions, and yellow represents the CA ligand.

There was no steric hindrance in the active sites of IL, as evidenced by the higher *V*_max_ for IL compared to free lipase (Table 1). CA has been shown to have very little diffusional resistance,^29,30^ which might explain the nonaffected enzyme–substrate reactions after immobilization of lipase. Also, a higher affinity was noticed toward the substrate in IL. Good affinity was further supported by docking scores reported in Figure 2a,b. Polar bond lengths of 3–3.8 Å were observed for IL using PyMOL software. IL unveiled higher *V*_max_ values than free lipase, confirming its enhanced catalytic activity. This is favorable for production of stable polymer films under the storage condition while accelerating the degradation process in a disposal environment. We here theorize that interactions between the lipase binding site (Figure 2a) and the CA matrix might create a CA-covered active site, thus achieving thermally stable IL without introducing any recombinant technology having higher catalytic latency for cellulose-based ester degradation. It has been previously described^15^ that controlling the active sites in proteinase K and its interactions between a matrix create an active site covered by the matrix, which modulated the enzyme partaking higher processive latency.

**Table 1:** Kinetic Parameters *V*_max_ and *K*_m_ Calculated from the Lineweaver–Burk Plot for Free Lipase and IL

pNPB	free lipase	IL
*V*_max_ (μmol min^–1^ mg^–1^)	0.2616	0.4151
*K*_M_ (mM)	81	11
*K*_cat_ (s^–1^)	8.75	1.4
(s^–1^ mM^–1^)	0.10	0.13

### Enzymatic Degradation of Films

Enzymatic degradation experiments were carried out under static conditions because polymer and oligomer chains can leach into an aqueous medium when the samples are shaken. Therefore, the static experiments can more clearly show the influence of enzymes on polymer degradation. First, to investigate the triggering effect of the embedded enzymes, CA, CA/IL 3%, and CA/IL 5% were incubated in a PBS buffer solution containing lipase from *C. rugosa* and cellulase from *A. niger*. The pH of the aging medium was maintained at 7.4. FTIR and TGA analyses were performed on the test samples before and after 7 and 40 days of aging to follow the changes in the chemical structure and thermal degradation behavior caused by the enzymatic degradation. Before analysis, the films were properly rinsed with distilled water and dried in an oven at 60 °C overnight.

**Figure 3:**
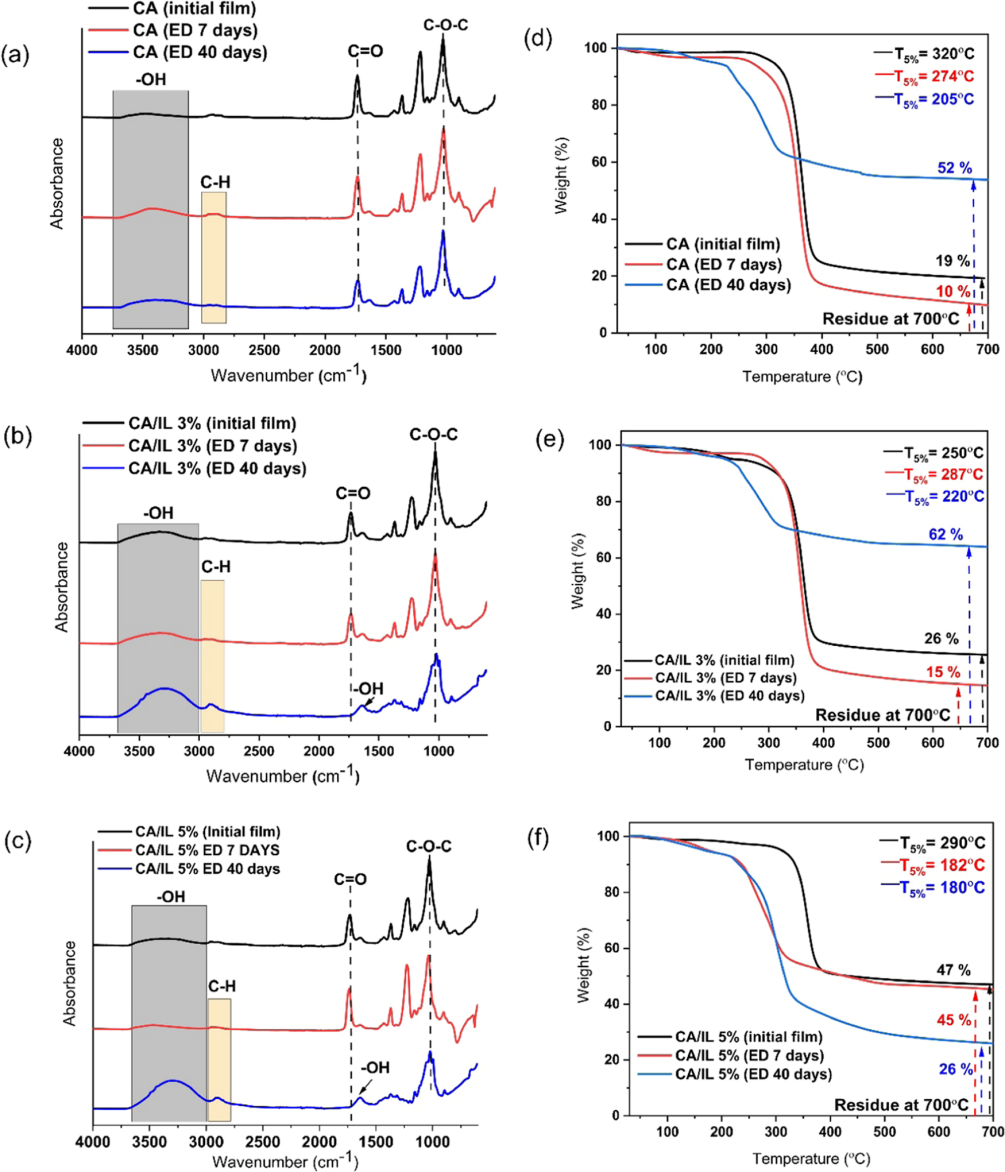
(a–c) FTIR spectra of CA, CA/IL 3%, and CA/IL 5% before and after enzymatic degradation and (d–f) TGA thermograms of the same samples before and after enzymatic degradation.

The FTIR spectra in Figure 3a–c demonstrate clear changes in the chemical structure, in particular for CA/IL 3% and CA/IL 5% with embedded enzymes. Figure 3a shows the FTIR spectra of plain CA originally and after 7 and 40 days of aging in an enzyme containing buffer solution. The spectrum after 7 days is almost identical to the original CA spectrum, and even after 40 days, only minor changes are observed; the intensity of acetate C=O at 1731 cm^–1^ slightly decreased in relation to cellulose C–O–C at 1034 cm^–1^, indicating some minor deacetylation. This is further supported by the slight increase in the intensity of the −OH band at 3600–3200 cm^–1^. In comparison, the spectra of CA/IL 3% and CA/IL 5%, shown in Figure 3b,c, had significantly changed after aging for 40 days in the enzyme containing buffer solution. The C=O band was no longer visible due to deacetylation, which is further supported by the increased intensity of the hydroxyl absorption band at 3600–3200 cm^–1^. In fact, the recorded spectra are very similar to the spectra of cellulose, indicating significant deacetylation. The deacetylation during the degradation experiments was further investigated by determining the remaining DS by NMR. Briefly, the original DS decreased from 2.2 to 1.7 for CA, from 1.90 to 1.20 for CA/IL 3%, and from 1.85 to 0.74 for CA/IL 5% during 40 days of enzymatic degradation. This corresponds to 23, 37, and 60% reduction in the degree of substitution for CA, CA/IL 3%, and CA/IL 5%, respectively. This clearly demonstrates the significant catalytic effect of embedded enzymes on deacetylation. There is also a clear correlation with the amount of added IL. The values also show that some deacetylation took place already during the preparation of the IL-containing films. DS before and after degradation in different environments as well as part of ^1^H NMR spectra in the region 1.8–2.2 ppm are shown in Figure S4.

The initial degradation onset, as determined by *T*_5%_, decreased by 30–70 °C after addition of IL (Figure 3d–f). This could be a result of the lower stability of IL (Figure 1c) and the reduced molecular weight (*M*_n_) and partial deacetylation during film preparation. The −NH functionality in the lipase could also contribute to the lower thermal stability of CA with IL. In earlier studies, deacetylation, cellulose chain scission, and the release of substances such as acetic acid, water, carbon dioxide, acetyl derivatives, and furans have been shown to take place during the thermal decomposition of CA.^31,32^ The thermal degradation behavior of CA and CA/IL 3% followed a similar trend after the enzymatic degradation. After 7 days, the TGA curves were very similar to the original curves, although a reduction in the *T*_5%_ temperature and a slight decrease in the amount of residue at 700 °C were observed. When the aging time was prolonged to 40 days, significant changes were observed in TGA curves and the *T*_5%_ temperatures dropped to 205 and 220 °C for CA and CA/IL 3%, respectively. The *T*_5%_ value of CA/IL 3% is close to 218 °C, which has been previously recorded for regenerated cellulose.^33^ Deacetylation is also supported by FTIR and NMR analyses as shown in Figures 3b and S4. Another significant change was the increase in the amount of residue from 10% after 7 days to 52% after 40 days for CA and from 15 to 62% for CA/IL 3%. In earlier studies, increased residues after thermal degradation of CA were reported both after crosslinking^32^ and after molecular weight reduction and deacetylation.^5,33^ Amine groups and inorganic compounds in IL might also facilitate carbonization.^34,35^

**Figure 4:**
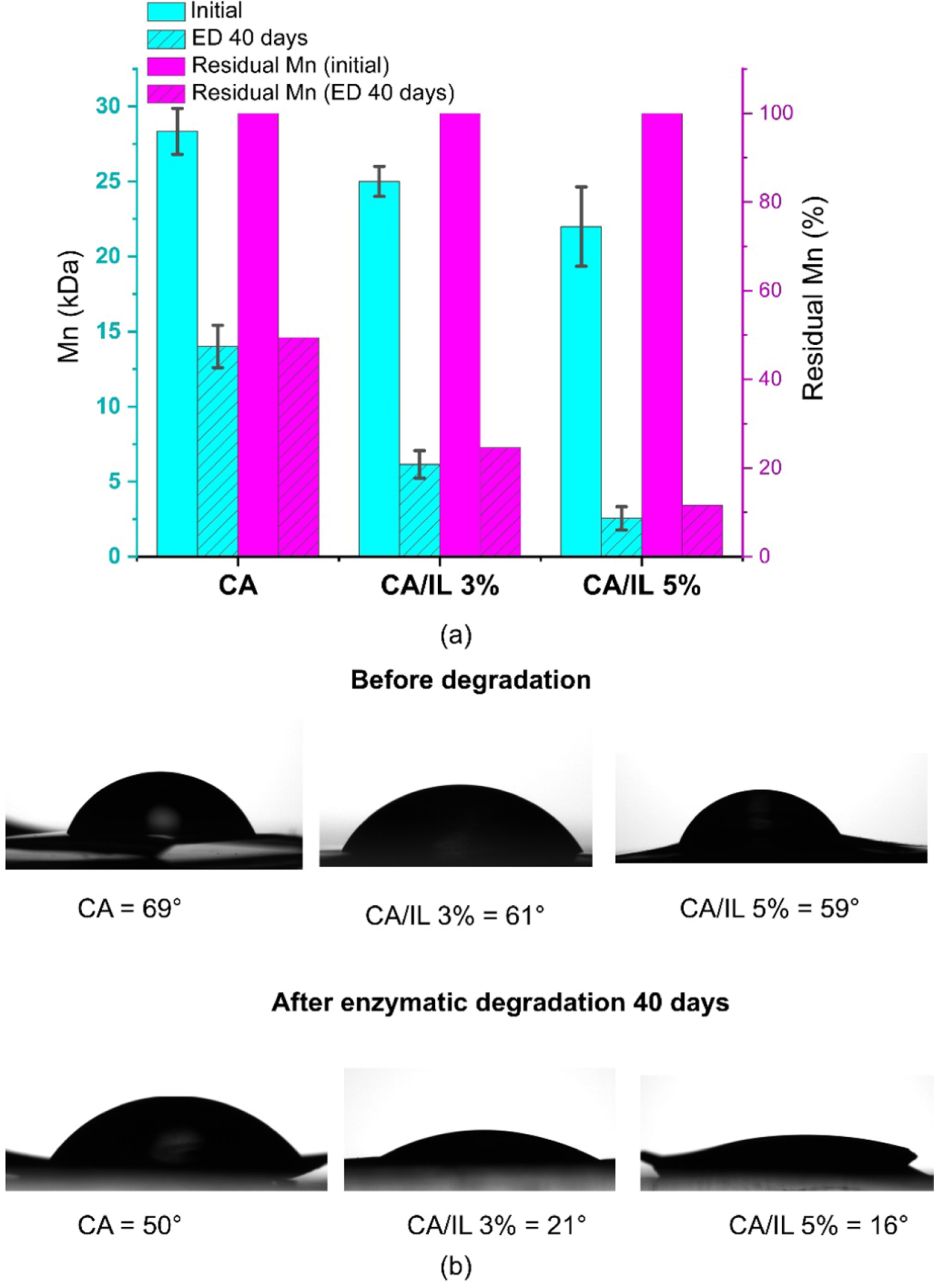
(a) Number-average molecular weight and residual *M*_n_ (% of original number-average molecular weight) of CA, CA/IL 3%, and CA/IL 5% before and after enzymatic degradation. (b) Water contact angles of the samples before and after enzymatic degradation.

While CA and CA/IL 3% followed a similar trend with aging time, a different behavior was observed in the case of faster degrading CA/IL 5%. Initially, increasing the IL constituent increased the residue at 700 °C from 19% for CA and 26% for CA/IL 3% to 47% for CA/IL 5% (Figure 3e,f). Due to the faster enzymatic degradation of CA/IL 5%, the TGA curve obtained after 7 days of enzymatic degradation resembled those recorded for CA and CA/IL 3% after 40 days of enzymatic degradation, indicating a higher degree of degradation. The degradation onset, *T*_5%_, had decreased to 182 °C, and the residue was 45%. *T*_5%_ remained similar when the degradation time was increased to 40 days, but the residue had decreased to 26%. According to the SEC results, the remaining material mainly consisted of oligomeric products (Figure 4), which might lead to evaporation instead of carbonization, resulting in a lower residue. In addition, it is likely that IL and inorganic compounds that might support carbonization had been released to the aging medium.

Figure 4a and Table S2 show that the molecular weight of the enzyme-embedded CA films decreased already during the film preparation, and it further decreased drastically during the 40 days of enzymatic degradation. This indicates that both deacetylation and chain scission by enzymatic hydrolysis took place.^36^Figure 4a also presents the percentage reduction of *M*_n_ with respect to the original molecular weights after film preparation. The most degraded sample, CA/IL 5%, underwent a significant 88% reduction in *M*_n_ during the 40 days of enzymatic degradation. However, CA and CA/IL 3% underwent 48 and 73% reduction of the original *M*_n_ during the same time period. The deacetylation and reduction of molecular weight are also expected to increase the hydrophilicity of the films due to the increasing number of free hydroxyl-groups. This is clearly illustrated by the water contact angles of the samples (Figure 4b). As an example, the contact angle of CA/IL 5% decreased from 59° to only 16° after 40 days of enzymatic degradation, showing a significant increase in hydrophilicity, in agreement with the recorded FTIR spectra. During the same time period, the contact angle of plain CA decreased from 69 to 50° in correlation with the significantly lower deacetylation and degradation rates. The observed molecular weight reduction indicates that the cellulase enzymes in the solution can cleave at least some of the 1,4-β-d glucosidic linkages, and this reaction is facilitated by the initial deacetylation reaction by immobilized lipases endo-cleaving the ester groups.^37,38^

**Figure 5:**
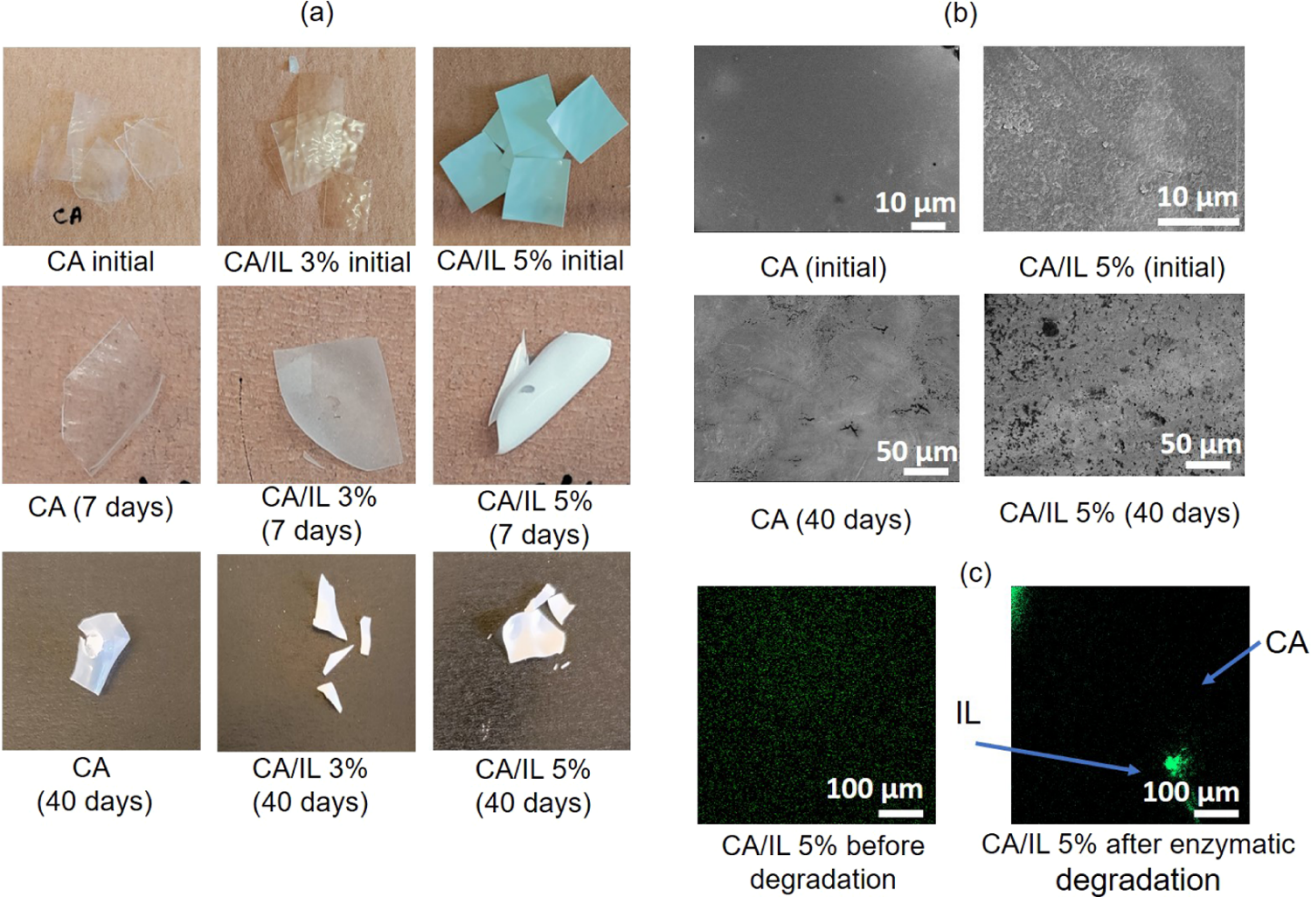
(a) Photographs of CA, CA/IL 3%, and CA/IL 5% films originally and after enzymatic degradation, (b) FESEM images of the samples before and after degradation and (c) dispersion of FL in the CA matrix originally and after 7 days of enzymatic degradation.

The degradation process is also visually evident from Figure 5a,b showing the photographs and FESEM images of the films before and after degradation. It is clear that the number of holes and cracks in CA/IL 5% is extensive after 40 days of enzymatic degradation, while only few minor holes are observed on plain CA without embedded enzymes. Moreover, to probe the enzyme distribution and presence in the films, lipase was fluorescently labeled (FL) and embedded in CA. CLSM was performed, showing that the enzyme still remained embedded in the CA matrix after 7 days (Figure 5c). A similar stability was earlier observed for lipase embedded in polycaprolactone (PCL) films.^15^ Lipase is known to act through covalent catalysis^39^ and for showing hydrolytic activity,^40^ which can deacetylate CA. Deacetylation or hydrolysis occurs when the substrate is temporarily covalently attached to the enzyme. The enzyme remains attached to the substrate throughout the enzymatic reaction, after which the bond is broken and the enzyme is regenerated. Over time, it becomes difficult to remove salts, ions, and enzymes present by the washing and drying process, which can increase the mass of the degrading material.^41^ This cleavage of bonds also creates cavities on the polymer and facilitates diffusion and adsorption of enzymes and other products formed during degradation.

### Biodegradation under Simulated Composting Conditions

**Figure 6:**
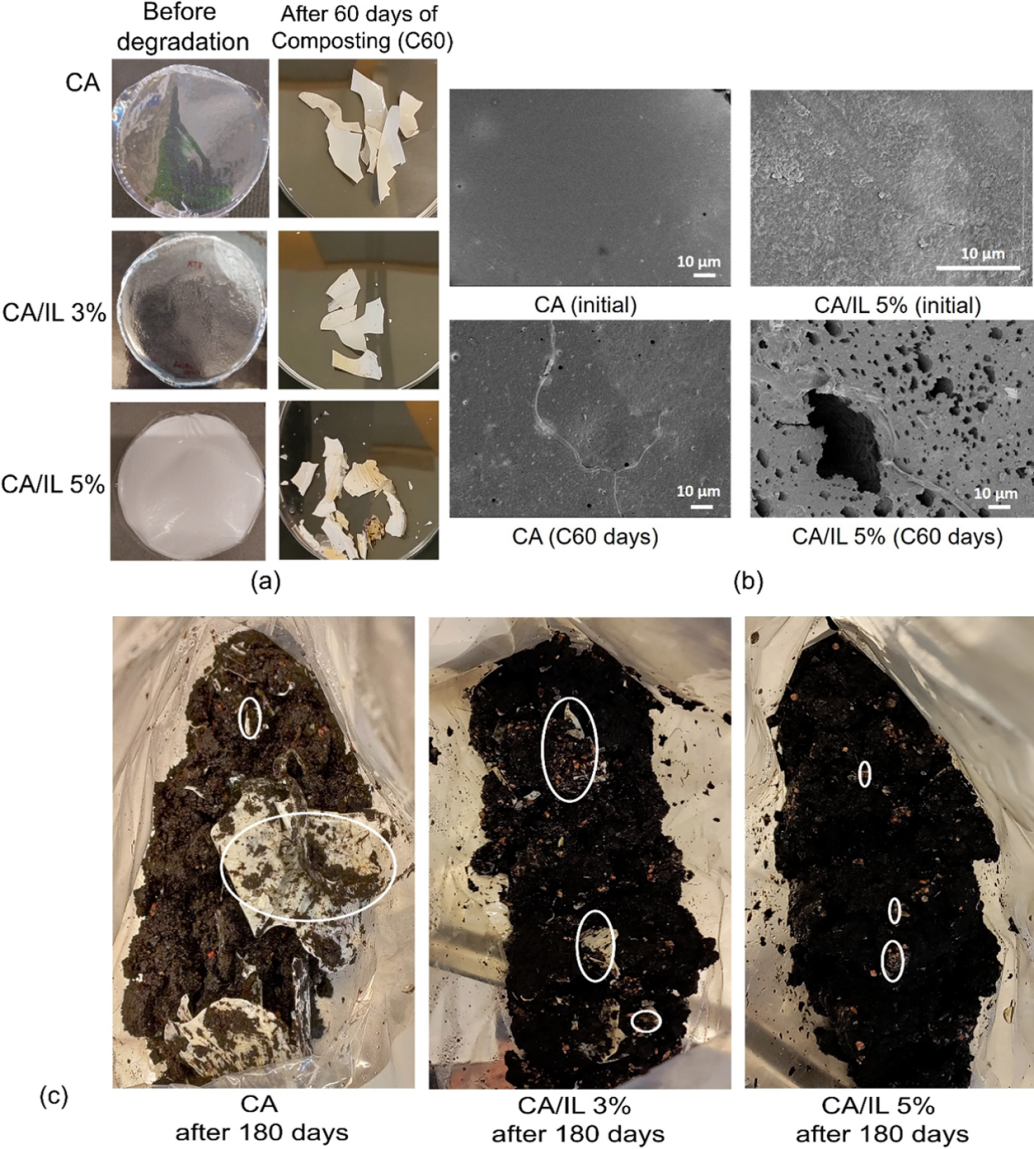
(a) Photographs and (b) FESEM images of CA, CA/IL 3%, and CA/IL 5% samples before and after simulated composting (c) soil and sample (marked in after 180 days of composting).

CA, CA/IL 3%, and CA/IL 5% films were also subjected to aging under simulated composting conditions at 58 ± 2 °C to understand their (bio)degradation behavior and the influence of embedded enzymes on the degradation process. The simulated composting experiments were performed for 60 days in order to evaluate the physicochemical property changes caused by the degradation process. Various studies^2,9^ suggest that the biodegradation of CA with a high degree of substitution (DS > 2) is significantly prohibited compared to plain cellulose or CA with DS < 2. Hence, we were interested to know if our enzyme-embedded approach would open a new window to faster degrading CA. The visual observations indicated increased opacity for all of the samples during the simulated composting (Figure 6a) with no other major differences. Hence, FESEM analysis was performed to further investigate the possible influence of the embedded enzymes on the morphology of the samples. From the FESEM image (Figure 6b), it is clear that the embedded enzymes significantly increased the formation of micrometer-scale holes in the CA/IL 5% samples. This further increases the possible sites for the microbial action to facilitate further deacetylation, intermediate formation, and mineralization.^41,42^ The significant cavities formed in CA/IL5% with embedded enzymes suggest that the degradation proceeded parallelly both on the surface and in the bulk, which was not reported earlier for CA-based samples. In the case of plain CA samples, only minor surface wear, tear, and cracks were observed (Figure 6b). Figure 6c presents photographs of the soil and remaining samples after 180 days of composting. The visible amount of sample remaining clearly illustrates the significant difference in the degradation rate. CA/IL 5% had degraded almost completely with only minor pieces remaining, while clearly visible large pieces of CA still remained. This signifies the effectiveness of the enzyme-embedded approach to facilitate the compostability of CA-based films.

For future studies, it would be of interest to study the enzyme conformation in the enzyme-embedded films to understand the closing and opening of the lids responsible for substrate binding. The embedded enzymes seem to remain embedded within the polymer matrix during the initial degradation period under composting conditions. The formation of a microbial biofilm or the adsorption of moisture and soil on the polymer surface during the degradation process changes the system from a closed-loop to an open-loop system.^43,44^ This allows substrates to enter the immobilized matrix, which may trigger the active sites of the lipase,^45^ leading to accelerated catalytic activity under composting conditions.

**Figure 7:**
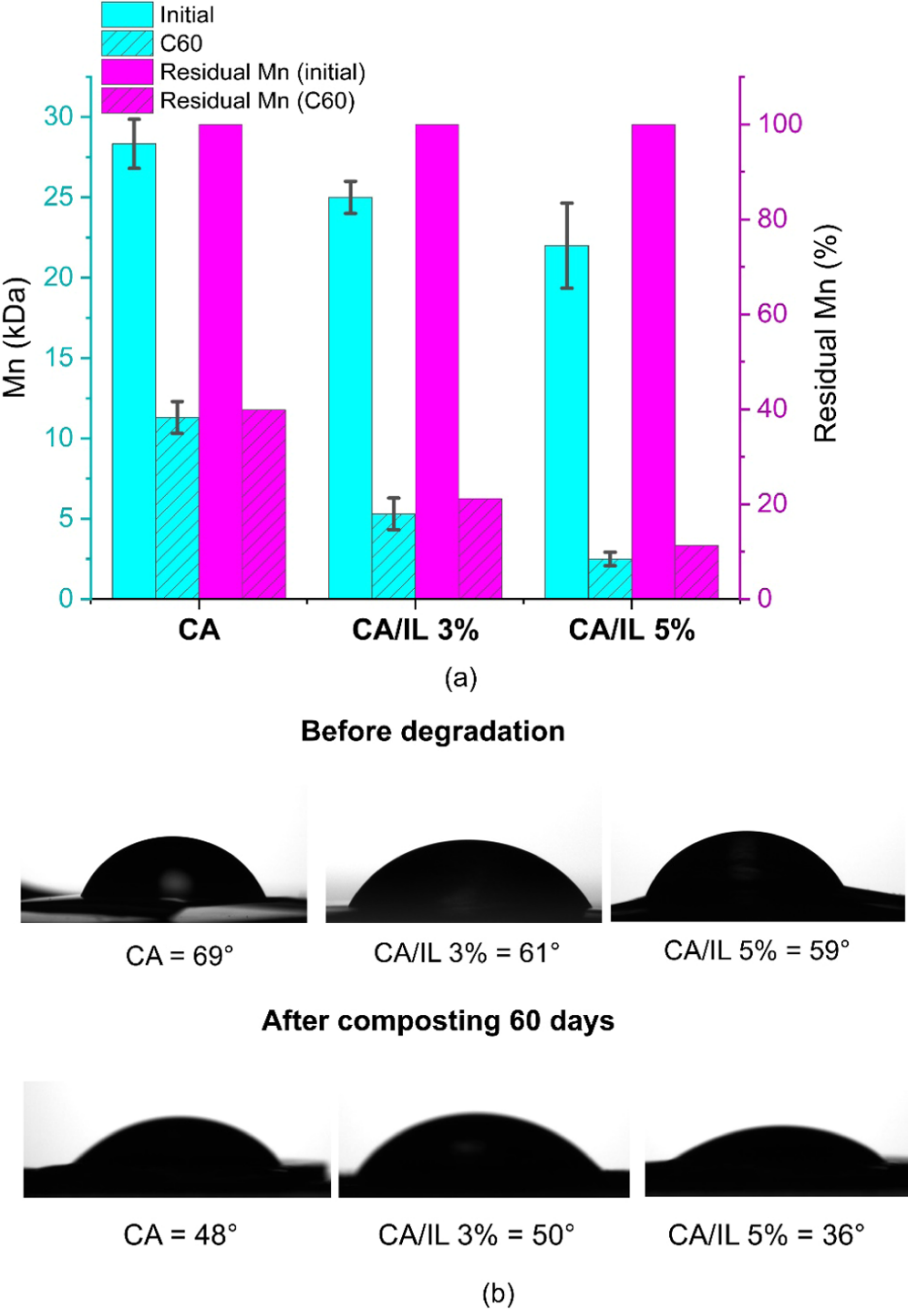
(a) Number-average molecular weight and residual *M*_n_ (% of original molecular weight) of CA, CA/IL 3%, and CA/IL 5% samples before and after simulated composting. (b) Water contact angle of the samples before and after simulated composting.

The degradation-triggering effect provided by the embedded enzymes is also evident from the molecular weight changes demonstrated after 60 days of simulated composting as presented in Figure 7a and Table S2 and the WCA analysis presented in Figure 7b. Simulated composting of the enzyme-embedded CA/IL 5% films leads to a decrease of 89% in *M*_n_ after 60 days, while a 60% reduction was recorded in the *M*_n_ of plain CA. This illustrates that all of the films were significantly degraded, but it also clearly demonstrates that the embedded enzymes accelerated the degradation of CA/IL 5% under composting conditions. Molecular weight analysis was also performed on the samples remaining after 180 days of composting (Table S3). After 180 days, CA/IL 5% had degraded almost completely and the remaining minor pieces had a very low *M*_n_ of 0.4 kDa, while the large pieces of remaining CA still had a relatively large *M*_n_ of 9.8 kDa.

This accelerating effect is even more significant considering the much larger weight loss for CA/IL 5%, based on the SEM images illustrating the highly porous structure for CA/IL 5% after composting. It should be kept in mind that the molecular weight, FTIR, NMR, and WCA analyses are performed on the remaining solid sample, and the actual differences in the degree of degradation based on, e.g., the visual differences in SEM images are expected to be even larger. Figure 7b presents the WCA of the samples after simulated composting. A significant decrease in WCA was observed for all of the composted films, but the decrease was even larger for CA/IL 5%, further supporting the faster degradation of the enzyme-embedded CA films.

**Figure 8:**
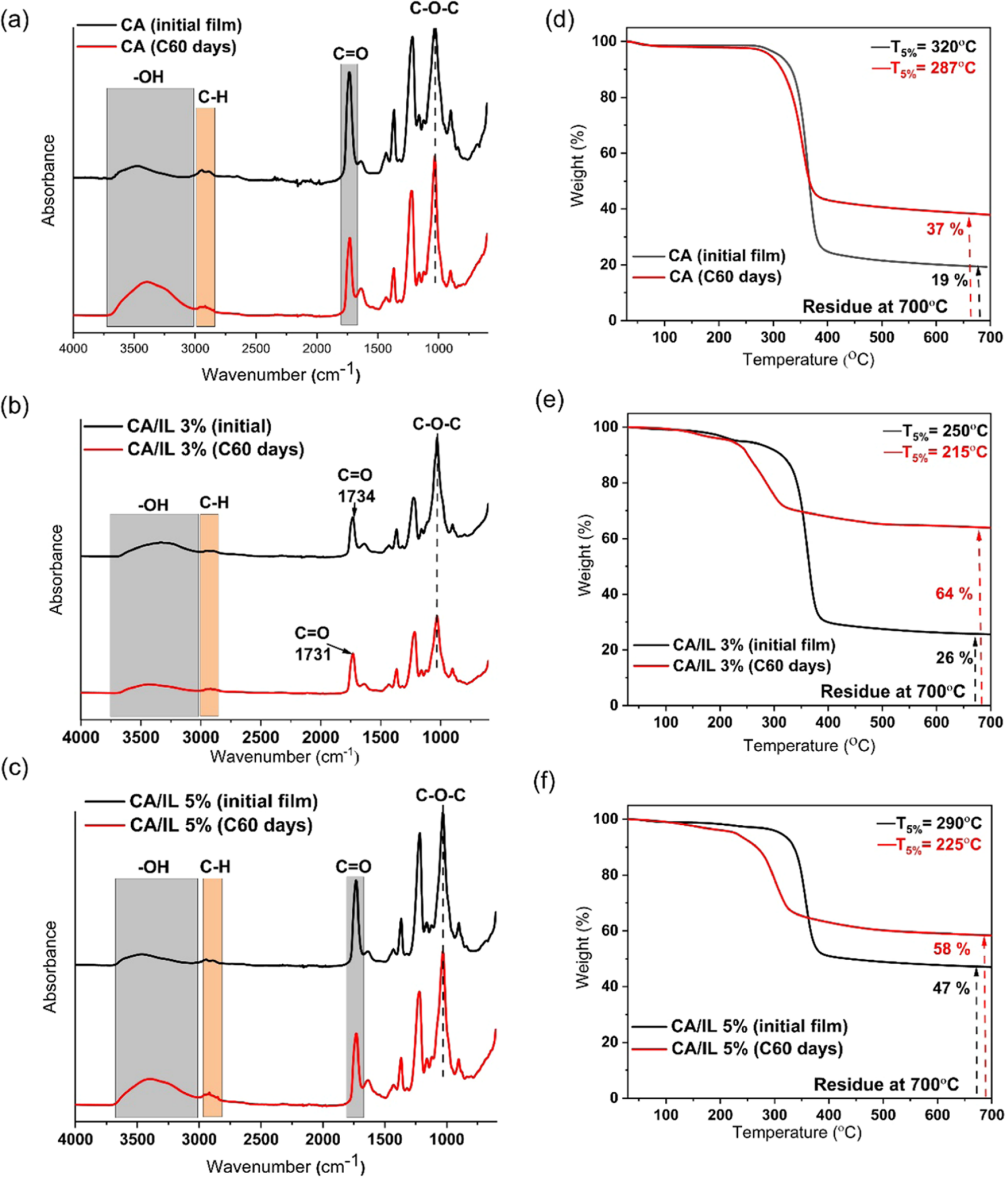
(a–c) FTIR spectra of CA, CA/IL 3%, and CA/IL 5% films and (d–f) TGA thermograms of the same samples before and after simulated composting.

The degradation process was further investigated by FTIR (Figure 8a–c) and NMR (Figure S4). We can observe that the FTIR spectra demonstrated significant changes after 60 days of simulated composting. Especially, the broad hydroxyl peak at 3500–3200 cm^–1^ increased in intensity, which could be attributed both to the deacetylation reaction and to the opening of the glycosidic bonds in the main chain of CA. Deacetylation was further supported by the shifting and decreased intensity of the carbonyl ester peak in the FTIR spectra. This was further confirmed by NMR analysis showing that the DS had decreased from 2.2 to 1.8 for CA, from 1.9 to 1.2 for CA/IL 3%, and from 1.85 to 0.9 for CA/IL 5%. This corresponds to 18, 37, and 51% reductions, respectively, after 60 days of composting. CA/IL 3% and CA/IL 5% samples composted for 180 days were no longer soluble in DMSO-*d*_6_, likely due to significant deacetylation, and could not be analyzed by NMR.

The higher IL content in the CA-based films initially decreased the onset of thermal degradation. After 60 days of composting, the onset of degradation had slightly further decreased for CA films, while a more significant decrease was observed for the more degraded CA/IL 3% and CA/IL 5% with embedded enzymes. The remaining residue at 700 °C for the original films was 19% for CA, 26% for CA/IL 3%, and 47% for CA/IL 5%, showing that the embedded enzymes promoted carbonization and char formation. The residue increased for all of the materials after 60 days of composting, but again the increase was significantly larger for the films with embedded enzymes and a higher degree of degradation. This again supports the fact that embedded enzymes accelerate the degradation of CA during composting (Figure 8d–f).

### Chemical Hydrolysis of the Enzyme-Embedded Films

Hydrolytic stability of the prepared films was followed for 180 days at 37 °C in pure water to see how stable the enzyme-embedded films are in contact with abiotic aqueous environments. In this case, we chose to only compare CA/IL 5% with CA since CA/IL 5% showed the fastest degradation both in the aqueous medium with the enzyme and during composting. This test validated that enzyme-embedded CA films can be used for short-term applications in contact with neutral aqueous environments at temperatures at least up to 37 °C. Polymer matrix compatibility with the enzyme immobilization matrix can, therefore, contribute to the design of enzyme-embedded films, where degradation is triggered when the materials come in contact with the catalytic medium without the need for any nondegradable or metal framework components. This polymer–enzyme–ILM compatibility and random substrate binding by the enzyme with the immobilization matrix can promote stability of the enzyme-embedded films in real applications.

**Figure 9:**
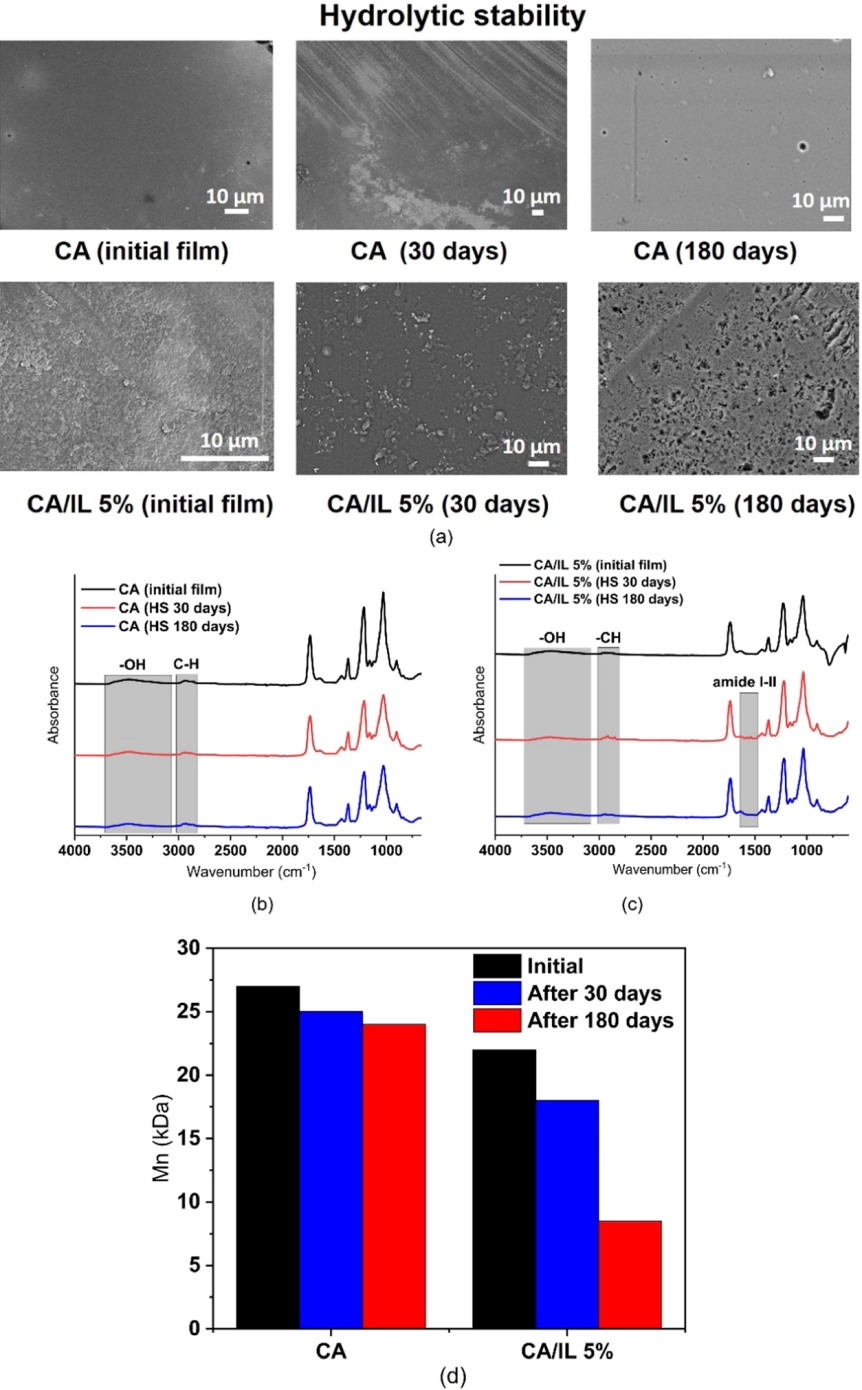
(a) FESEM images, (b, c) FTIR spectra, and (d) number-average molecular weight of CA and CA/IL 5% before and after the hydrolytic stability test performed in water at 37 °C.

From Figure 9a, we can observe that there were no significant surface changes in CA and CA/IL5% films after 30 days of hydrolytic aging. This is further supported by the FTIR spectra in Figure 9b,c, illustrating similar spectra to the original. After 180 days, only minor cracks were observed on the surface of CA films, while more holes and cracks had appeared in the CA/IL 5% films. The WCA of CA decreased from 69 to 52°, while the WCA of CA/IL 5% decreased from 59 to 41° (Figure S5). The observed decrease in WCA agrees with the reduction of DS from 2.2 to 1.85 in the case of CA and from 1.85 to 1.30 in the case of CA/IL 5% as determined by NMR (Figure S4). Minor deacetylation has earlier been shown to take place through chemical hydrolysis of the acetylate groups during aging in abiotic aqueous environments.^46^ Deacetylation was clearly promoted by the embedded enzymes in CA/Il 5% although the rate was slower in water compared to the aqueous medium containing enzymes. From Figure 9d and Table S4, it is clear that in pure water, neat CA is quite stable even up to 180 days, which has also been seen in previous studies.^46,47^ There are no major changes in the FTIR spectra, and only a minor decrease of *M*_n_ is observed. CA/IL 5% was relatively stable during the first 30 days with a *M*_n_ decrease from 22 to 18 kDa. However, after 180 days, the *M*_n_ of CA/IL 5% had decreased to 8.5 kDA in comparison to CA, which still had a *M*_n_ of 24 kDA. The degradation process might be further enhanced by leaching of the embedded enzymes from the films leaving behind cavities, which was also observed in Figure 9a. Further, these cavities facilitate water diffusion and could contribute to further weight loss and molecular weight reduction. The changes in FTIR spectra were not as significant. An increase in peak broadness at 3500–2900 cm^–1^ is observed, which could be due to deacetylation and/or water absorption (Figure 9b,c). This indicates that even the enzyme-embedded materials could be used in contact with an aqueous medium for shorter periods of time.

In conclusion, our results correlate with previous literature that demonstrated the crucial role of DS and deacetylation for the enzymatic degradability and biodegradability of CA. One example is the ability of cellulase enzyme to degrade randomly substituted 2,3-O-CA of DS 0.4–1.3 within 30 min of incubation at pH 5,^48^ whereas the same material of DS = 2.0 displayed no degradability. Similarly, Ishigaki et al.^49^ reported that a combination of bacterial lipase from Bacillus sp. S2055 and cellulase was able to break down a plastic sheet of CA having a DS value of 1.7. Another study reported breakage of CA films with DS = 1.7 when exposed to cellulase enzyme media for 20 days; however, they reported no degradation of CA (DS = 2.4).^12^ Reportedly, at least partial deacetylation of CA with high DS is required for the degradation to proceed at a reasonable rate via scissions of ″regenerated″ cellulose chains.^50^ Our enzyme-embedded approach can bring CA materials under the threshold of adequate deacetylation (DS) to facilitate the follow-up degradation process.

## Conclusions

Embedded enzymes effectively catalyzed the deacetylation and degradation of CA films during aging in an aqueous solution with external enzymes and during simulated composting experiments. A method was developed to physically entrap lipase in CA particles, which were further incorporated in CA films. By using CA as the entrapment matrix, the biobased nature of the material was retained and addition of nondegradable components was avoided. The immobilization increased both the thermal stability and catalytic activity of the enzyme. When comparing the degradation of CA and CA/IL 5% with embedded enzymes, significant acceleration of the degradation rate was observed. The enzyme-embedded strategy promoted degradation in both the surface and bulk of the material, forming microscale cavities that were not observed in CA films without enzymes. The significant degradation accelerating effect was further confirmed by molecular weight changes and chemical changes, especially deacetylation, illustrated by FTIR, NMR, and WCA. Furthermore, aging of the films in pure water showed satisfactory stability for several days. This enzyme-embedded polymer degradation approach is expected to be transferrable to other polymer systems, such as other chemically modified lignocellulose materials, to retain their inherent biodegradability and substantial material stability.

## Supporting Information Available

Immobilization ratio and immobilization efficiency of IL, molecular weight before and after degradation, NMR analysis of the degree of substitution before and after degradation, and water contact angle images of the samples before and after degradation (PDF)
