# Supplementary material for: Triggering Degradation of Cellulose Acetate by Embedded Enzymes: Accelerated Enzymatic Degradation and Biodegradation under Simulated Composting Conditions
Source: Biomacromolecules. 2023 Jun 22;24(7):3290–303. doi: 10.1021/acs.biomac.3c00337 (PMC10336969; doi:10.1021/acs.biomac.3c00337)
Supplement: Supplementary file 1 — bm3c00337_si_001.pdf [file bm3c00337_si_001.pdf]

Supplementary information

Triggering degradation of cellulose acetate by embedded enzymes: Accelerated enzymatic degradation and biodegradation under simulated composting conditions

Naba Kumar Kalita[1] and Minna Hakkarainen[1]*
KTH Royal Institute of Technology, Department of Fibre and Polymer Technology, Teknikringen 58, 100 44 Stockholm, Sweden

[*]Corresponding author email: minna@kth.se

This supporting information contains 4 tables and 5 figures on 6 pages**.**

Enzymatic activity measurements

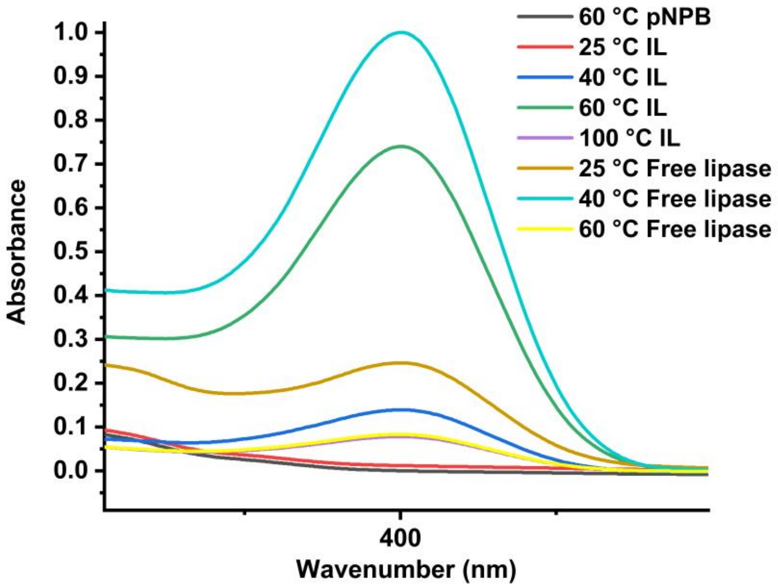

Figure S1. Thermal stability of IL and free lipase was measured recording the absorbance of its efficacy to hydrolyze pNPB.

Table S1. Immobilization ratio of the lipase in CA matrix

Concentration in (mg/mL)	(C_0)	C	V	W	I
0.002	0.191057	0.033383053	50	2026	$1.332\text{x}10^{-2}$
0.004	0.573057	0.019283053	50	2026	$4.239\ \text{x}10^{-3}$
0.0004	0.028057	0.012383053	50	2026	$3.868\ \text{x}10^{-4}$

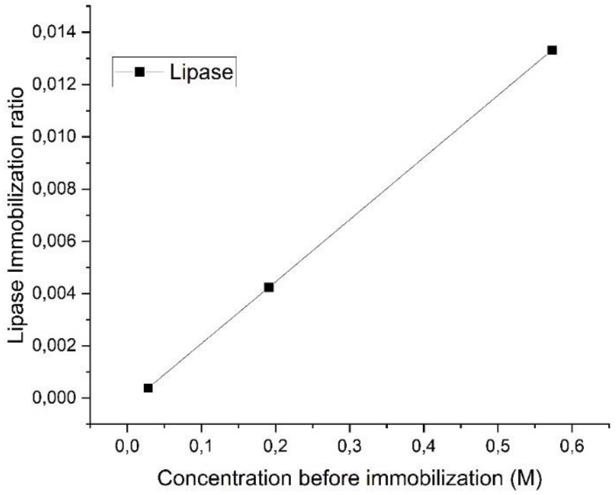

Figure S2. Immobilization ratio of lipase on CA matrix.

Enzyme activity (immobilization efficiency):

Immobilized lipase activity (3 g/L) = 27.55% and immobilized lipase activity (2 g/L) = 17.55%.

Increase in concentration of lipase displayed increase in enzyme activity.

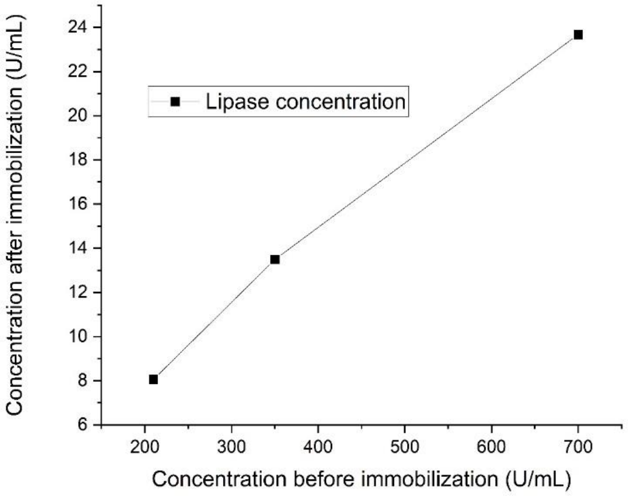

Figure S3: Efficiency of lipase before and after immobilization on CA matrix.

Degree of substitution by NMR

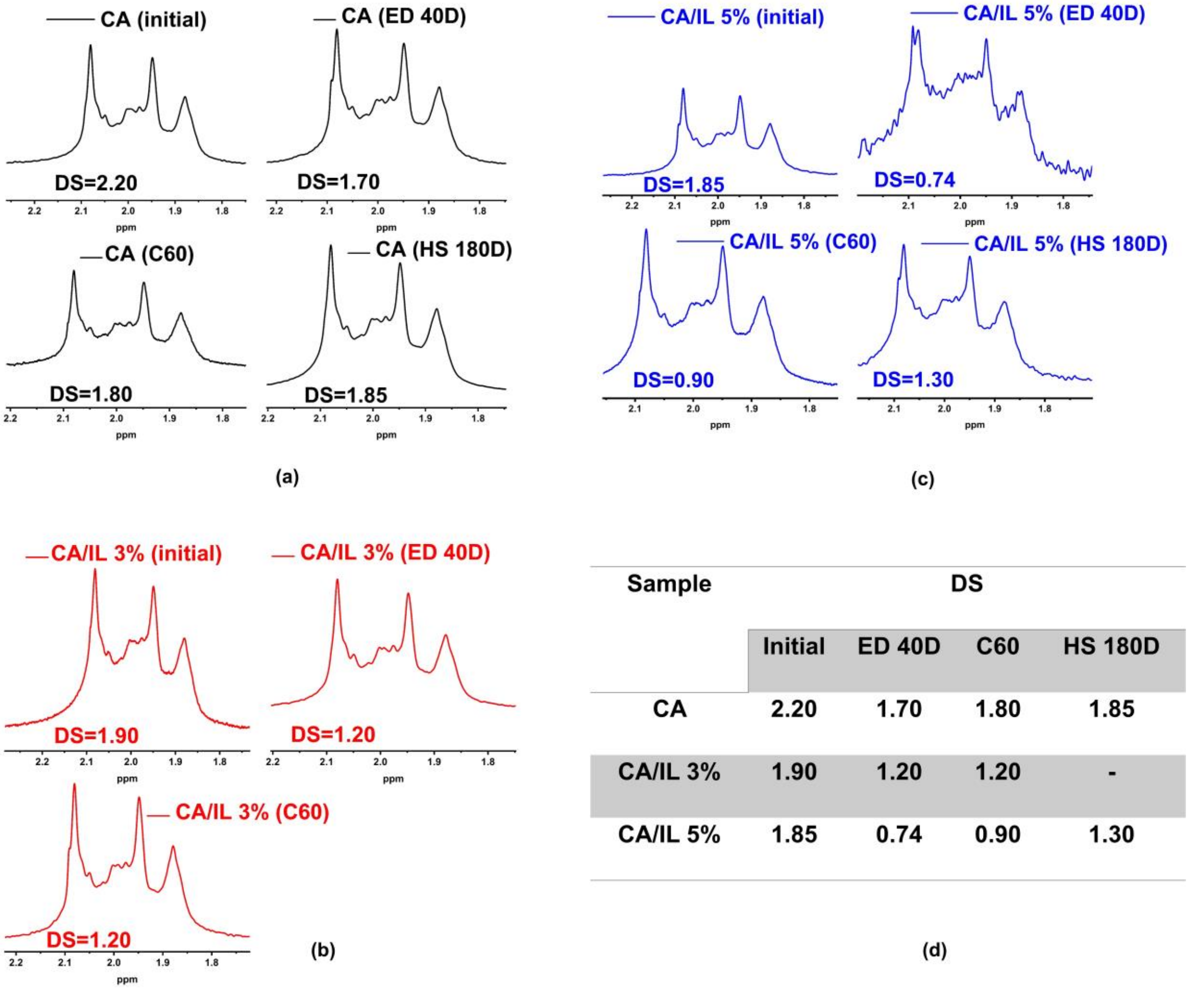

Sample	DS			
	Initial	ED 40D	C60	HS 180D
CA	2.20	1.70	1.80	1.85
CA/IL 3%	1.90	1.20	1.20	-
CA/IL 5%	1.85	0.74	0.90	1.30

Figure S4. ^{1}H NMR spectra showing the region between 1.8-2.2 for the acetyl-groups of (a) CA, (b) CA/IL 3% and (c) CA/IL 5% before and after aging in different environment and (d) a summary of the corresponding DS for the three materials before and after aging in different environments.

Molecular weight analysis

Table S2. Molecular weight analysis of test samples before and after degradation.

**Sample**	**Initial (kDa)**			**40 days of enzymatic degradation (kDa)**			**60 days of composting (kDa)**		
	M_n	M_w	*Đ*	M_n	M_w	*Đ*	M_n	M_w	*Đ*
**nCA**	28.3	76	2.7	15	15	1.0	10.5	10.6	1.0
**CA/IL 3%**	25.0	68	2.7	5.5	6.7	1.2	4.6	7.5	1.6
**CA/IL 5%**	22.0	72	3.2	2.0	2.1	1.0	2.2	2.5	1.2

Table S3. Molecular weight of the samples before and after 180 days of composting.

Sample	**Initial (kDa)**			**180 days of composting (kDa)**		
	M_n	M_w	*Đ*	M_n	M_w	*Đ*
CA	28.3	76	2.7	9.8	18	1.9
CA/IL 3%	25.0	68	2.7	0.8	2.1	4.2
CA/IL 5%	22.0	72	3.2	0.4	1.2	3.0

Water contact angle of the test samples subjected to hydrolytic stability test

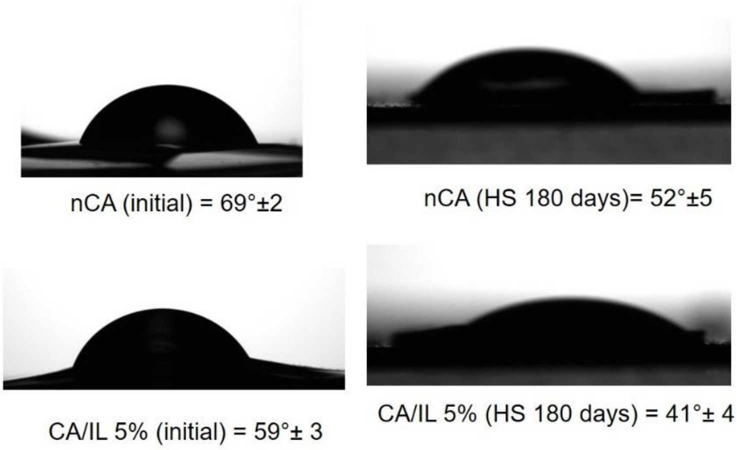

Figure S5. WCA of the test samples before and after hydrolytic stability test.

Table S4. Molecular weight before and after hydrolytic stability test.

**Sample**	**Initial (kDa)**			**30 days of hydrolysis (kDa)**			**180 days of hydrolysis (kDa)**		
	M_n	M_w	*Đ*	M_n	M_w	*PDI*	M_n	M_n	*Đ*
**nCA**	27	76	2.7	25	68	2.8	24	85	3.3
**CA/IL 5%**	24	72	3.0	18	41	2.3	8.5	9.2	1.1
